# Retraction Note: Circular RNA circ_0000517 regulates hepatocellular carcinoma development via miR-326/IGF1R axis

**DOI:** 10.1186/s12935-024-03341-1

**Published:** 2024-04-27

**Authors:** Shuwei He, Jianzeng Yang, Shitao Jiang, Yuan Li, Xingmin Han

**Affiliations:** Department of Nuclear Medicine, The First Affiliated Hospital of Zhengzhou University, No.1 Jianshe East Road, Zhengzhou, 450000 Henan China

**Retraction Note: Cancer Cell International (2020) 20:404**

10.1186/s12935-020-01496-1

The authors have retracted this article. After publication, the authors found that the cell lines used in the experiments presented in this article were contaminated with HeLa cervical cancer and unidentified mouse cells, which was confirmed by STR profiling. This undermines the results and conclusions of this article.

All authors agree to this retraction.

